# Experiences of deep concentration in individuals with attention deficit hyperactivity disorder (ADHD): An integrative review of the literature

**DOI:** 10.1192/j.eurpsy.2025.1885

**Published:** 2025-08-26

**Authors:** M. B. Terra, P. M. Etchebest

**Affiliations:** 1 Department of Internal Medicine; 2 Department of Psychology, Federal University of Health Sciences of Porto Alegre, Porto Alegre, Brazil

## Abstract

**Introduction:**

ADHD has been seen by current science as an expression of complex traits that go beyond the symptomatological triad of “inattention-hyperactivity-impulsivity”. Among the new notes, experiences of deep concentration (DC) are mentioned as a frequent phenomenon in the daily lives of those with ADHD, which encourages research on this topic.

**Objectives:**

The main objective of this integrative review was to investigate how empirical scientific studies have evaluated, associated and understood this hyperfocused attentional pattern in individuals with a diagnosis or symptoms suggestive of ADHD.

**Methods:**

For searches in the electronic databases PubMed, Scopus, LILACs, Pepsic and Scielo, the terms “attention deficit hyperactivity disorder”, “hyperfocus” and “flow state” were chosen. As inclusion criteria, studies were considered with (a) full texts available, (b) with an empirical design, (c) in English and (d) published in any year. In total, 10 empirical studies were analyzed (8 quantitative and 2 qualitative), Regarding the critical analysis of the included publications, relevant data were extracted about: (a) assessment instruments for deep concentration measures, (b) ADHD assessment instruments, (c) associations between hyperfocus, flow and ADHD and (d) general understandings of researchers on the representation of DC experiences in the lives of those with ADHD.

**Results:**

The sudies indicated a plurality of assessment instruments for both CP constructs (hyperfocus, flow, perseveration) and ADHD symptoms. As main findings, the articles suggest a high prevalence of hyperfocus in adults with ADHD compared to those without the disorder, as well as positive correlations between ADHD symptoms, hyperfocus, perseveration, internet addiction and emotional dysregulation. Different perspectives on the manifestation of DC in ADHD were captured from the studies, whose interpretations ranged from harmful behavior to the potentiality of ADHD.

**Conclusions:**

It was observed that, despite the recent expansion of research trying to understand the phenomenon of hyperfocus in the context of ADHD, scientific knowledge on the topic is still quite limited. In addition to having the unprecedented character of bringing together what modern science has postulated about CP in relation to ADHD, this research is relevant because it contributes to expanding the visibility of ADHD beyond the diagnostic criteria established by the DSM-V-TR and ICD-11.

**Disclosure of Interest:**

None Declared

